# Mortality from Pleural and Lung Cancer in Railway Maintenance Workers

**DOI:** 10.3390/life15071155

**Published:** 2025-07-21

**Authors:** Leonardo Scarso, Marco Novelli, Eva Lorenza Negri, Carlotta Zunarelli, the Pleural Cancer 2024 Study Group, Francesco Saverio Violante

**Affiliations:** 1 Occupational Medicine Unit, Department of Medical and Surgical Sciences, Alma Mater Studiorum, University of Bologna, Via Pelagio Palagi 9, 40138 Bologna, Italy; leonardo.scarso2@unibo.it (L.S.); eva.negri@unibo.it (E.L.N.); francesco.violante@unibo.it (F.S.V.); 2 Department of Statistical Sciences, Alma Mater Studiorum, University of Bologna, Via Belle Arti 41, 40126 Bologna, Italy; m.novelli@unibo.it; 3 Division of Occupational Medicine, IRCCS Azienda Ospedaliero-Universitaria di Bologna, Via Pelagio Palagi 9, 40138 Bologna, Italy

**Keywords:** asbestos, lung cancer, smoke, railway carriage maintenance workers, occupational exposure

## Abstract

(1) Background: Occupational exposure to asbestos remains a significant public health concern due to its association with pleural cancer and other cancers. This cohort study examines the incidence of asbestos-related diseases among railway carriage maintenance workers exposed to asbestos between 1960 and 1979 in Bologna, Italy. (2) Methods: A cohort of 2197 male workers was followed from 1960 onwards, with data collected on asbestos exposure, smoking habits, and mortality outcomes. The association of asbestos exposure and smoking with the risk of pleural cancer and lung cancer was assessed using Cox proportional hazards regression models. (3) Results: This study identified a substantial burden of asbestos-related pleural cancer, with an exponential increase in risk over time since the beginning of exposure. Our results suggest the lack of a multiplicative effect of asbestos exposure and smoking on lung cancer risk. The Cox models showed a significant association between smoking and lung cancer risk, with a hazard ratio of 3.26 (95% CI: 1.10–9.64, *p* = 0.03), less significant for asbestos exposure, with a hazard ratio of 1.42 (95% CI: 0.66–3.06). (4) Conclusions: This study provides valuable insights into the long-term health effects of occupational asbestos exposure and underscores the complex interaction between asbestos exposure and smoking in the development of lung cancer.

## 1. Introduction

The construction and maintenance of railway carriages was one of the several industries in which asbestos was used extensively in the last century. After the spread of knowledge about the extreme danger of exposure to this mineral, safer working methods were gradually used, up to the complete ban of the use of asbestos in several countries (in Italy, it took place in the early 1990s). Malignant mesothelioma is a very deadly cancer which requires strong attention to be paid to the relationship between exposure to asbestos both occupational and environmental.

Malignant mesothelioma of the pleura and peritoneum is certainly the distinctive form of tumor from exposure to asbestos; the latter, however, can also cause tumors in the lungs and other organs [1]. Although malignant mesothelioma is a rare tumor in people not exposed to asbestos [2], in workers who incur this exposure, it can cause a considerable number of deaths: in the past, in the most exposed cohorts, up to 10% or more [3].

Malignant mesothelioma from exposure to asbestos has been the subject of several epidemiological studies which have highlighted some peculiar characteristics, compared with all other known carcinogens, such as the increased risk of disease as a function of the time elapsed since the first exposure, even after this has ceased. Regarding the relationship between exposure to asbestos and the development of malignant mesothelioma, there remain a few points that still seem to require a complete definition:

- Whether the risk of malignant mesothelioma in individuals who have been exposed to asbestos continues to increase indefinitely with the time elapsed since the first exposure or ceases to increase after a certain time;
- What the minimum latency of malignant mesothelioma from the start of exposure to asbestos is.

Also, in the case of lung cancer in subjects exposed to asbestos, some points remain awaiting a complete definition:

- Whether there is (and what type it is) an interaction between cigarette smoke and exposure to asbestos in the determinism of lung cancer; the first studies on this topic seemed to suggest a multiplicative interaction [4], while the most recent ones seem to suggest a simple additive effect [5].
- If the risk of lung cancer decreases after stopping exposure to asbestos [6,7], or if, as in the case of cigarette smoking, it simply stops increasing [8].

Thus, the main aim of this study is to elucidate the relationship between lung cancer and asbestos exposure and cigarette smoke in terms of the effect of these risk factors combined on cancer occurrence.

To answer some of these questions, we started a cohort study on mortality due to malignant mesothelioma and lung cancer in a group of workers formerly employed in the maintenance of railway carriages, for some of whom there was information on smoking habits.

## 2. Materials and Methods

The Institutional Review Board of the University of Bologna (number 205120, 12 September 2019) approved the study protocol.

The workers in this study were employed in a railway carriage maintenance company in Bologna, Italy.

Based on information published in the past and some reports on exposure to asbestos in that company, it was possible to define that the period of greatest use of this mineral began in the early 1960s, while in the second half of the 1970s, preventive measures were implemented, which were deemed effective. Consequently, we defined as a period of interest for this study the interval between 1 January 1960 and 31 December 1979 (20 years).

A published article [9] summarizes a report from the Local Health Authority dated June 1981, in which the level of exposure was measured in the different areas of the company. The asbestos type used was mostly reported as crocidolite, with some amosite. Asbestos fibers were determined by means of phase contrast microscopy. Of course, in the period of interest, the exposure of the workers to asbestos could have been higher, given that the exposure data reported were measured only in 1980. Therefore, the results reported in that article may be used as an indirect indication of the real exposure during the period of interest.

We obtained from the company the list of workers, directly employed, who had worked in the plant for at least one day in the interval of interest; the list was reconstructed by the company based on paper documentation. On the other hand, no information was available about the workers who worked in that plant, in the period of interest, employed by other employers.

The different job titles during the employment period were available for each worker, but the measure of exposure to asbestos was not; thus, for the purpose of this study, we categorized the workers according to “white-collar” or “blue-collar” roles within the company. Individuals in “white-collar” roles may have been indirectly exposed to asbestos, while those in “blue-collar” roles likely experienced direct exposure, albeit in different measure. Considering the possibility of job transitions and changes in work settings, we allocate this classification for each worker based on the total duration spent in each category, either “white-collar” or “blue-collar”, throughout the whole observational period.

The living status of each worker was checked on 31 December 2022, through access to the electronic archive of residents in Italy. For subjects not found in this archive, a request for verification of alive status was sent to the municipalities of birth. For the deceased subjects, a request was sent to the Local Health Authorities where the registration of cause of death is kept, to ascertain the underlying cause of death.

During the 1990s, the corporate health service of the company requested to the employees who had previously worked in the company if they wished to undergo a health check. We searched paper medical records of workers who accepted to be checked and recorded all the information found in the smoking section of those records. Unfortunately, data about smoke were not reported for all employees; thus, we used multiple imputation by chained equations (MICE) to solve it. No other data about health status were extracted and analyzed.

Transition from ICD-9 to ICD-10 codes for the causes of death was mostly accomplished, in the different areas of the country, after 2005; consequently, we received ICD-9 codes for causes of death up to that period and ICD-10 after that time. A specific ICD code for mesothelioma (C45) was introduced with ICD-10 (1994); ICD-9 only contained a code for “pleural cancer” (163). It is worth noting that in our cohort, no ICD-10 code for “pleural cancer” (C38.4) was obtained for deaths occurring after 2009 (after that time, only codes for mesothelioma were obtained). For this reason, we decided to collect both ICD-9 “pleural cancer” and ICD-10 “mesothelioma” under a single category of “pleural cancer” for the purpose of this analysis.

It is also worth noting that after 2009, the diagnosis of mesothelioma in Italy can be considered much more accurate than before, due to the widespread use of immunohistochemistry; previously, some cases coded as pleural cancers may have included cancer of the pleura other than mesothelioma (for example, pleural metastases of other cancers).

To investigate the association between asbestos exposure and the incidence of pleural cancer, as well as lung cancer, with the addition of tobacco consumption behavior, Cox regression models [10] were adopted. They examined data pertaining to exposure categorization and smoking habits (where 1 was used to indicate smoker or past smoker status and 0 to indicate never-smoker).

Unfortunately, we did not perform standardization because it was not easy to find an external population with similar characteristics as our cohort.

The analysis of data was performed by means of R software, version 4.2.2 [11].

## 3. Results

The study cohort of 2200 individuals, predominantly male, included only 3 females, who were excluded from analyses. Of the remaining participants, 845 were alive (average age of 77.4 years), 1303 were deceased (average age of 73.7 years), and 49 had undetermined status. Among the 1303 deaths, 105 lacked information. A health check subset of 671 workers (30.5% of the cohort) provided smoking habit data, with 263 deceased (20.2% of all deaths).

Table 1 shows the temporal distribution of pleural cancers and mesotheliomas from 1960 onwards, with cases identified by ICD-9 code 163 and ICD-10 code C45. The first pleural cancer case in the cohort was recorded in 1979, and the first mesothelioma case in 1997. For this study, both are categorized as “pleural cancers.”

The relative risk of dying from pleural cancer for blue-collar workers (N = 2015) is 1.27 compared with white-collar workers (N = 146), with incidence rates of 0.90 and 1.15, respectively. This higher risk among blue-collar workers is expected due to their greater asbestos exposure, as even low levels pose significant risks. The job title is only an indirect estimate of asbestos exposure.

Blue-collar workers had a 9.2% (98/1065) death rate from pleural cancer, compared with 4.4% (5/114) among white-collar workers. The crude mortality ratio of 2.09 indicates that pleural cancer deaths are over twice as common among blue-collar workers. The incidence rate is 1.1 per 1000 person-years for blue-collar workers and 0.9 per 1000 person-years for white-collar workers, from the first exposure. After stopping exposure, the rates are 1.3 and 1.2 per 1000 person-years, respectively, again suggesting greater exposure among blue-collar workers.

The mean and median time to pleural cancer since exposure began were 39.5 and 40.9 years, respectively, ranging from 11.0 to 54.7 years. Since the end of exposure, the mean and median times were 29.2 and 30.5 years, respectively, ranging from 4.5 to 51.9 years.

**Figure 1:**
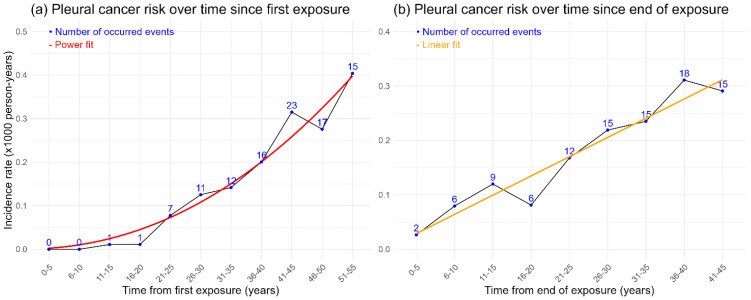
(**a**) Risk of pleural cancer (incidence rate per 1000 person-years) for each 5-year interval of time since first exposure. (**b**) Risk of pleural cancer (incidence rate per 1000 person-years) for each 5-year interval of time since the end of exposure.

Figure 1 shows the risk of pleural cancer over time. In Figure 1a, the incidence rate increases exponentially post-initial exposure, starting from 0 in the first 10 years and peaking at 0.40 per 1000 person-years in the 51–55-year interval. A power function fit indicates that the incidence rate amplifies by 2.15 times each interval (95% CI 1.68–2.72), showing a significant exponential increase.

In Figure 1b, the incidence rate rises over time after exposure ends, peaking between 36 and 40 years, with a linear trend suggesting increasing risk over time, though data variability is present.

A Cox proportional hazards regression model was used to assess exposure duration’s effect on pleural cancer risk. The hazard ratio for exposure duration is 0.97, suggesting a 3% decrease in the hazard of dying from pleural cancer per unit increase in duration. However, a *p*-value of 0.11 indicates insufficient evidence to confirm this effect.

Table 2 shows smoking status by firstly job title (662 subjects with complete data) and secondly life status (excluding 49 with unverified status). The prevalence of smoking was 40% in white-collar workers (8/20) and 40.7% in blue-collar workers (261/642). More never-smokers have died compared with smokers, with 70% mortality among those with unknown smoking status.

Table 3 shows the temporal distribution of lung cancers from 1960 onwards, with the first case recorded in 1966.

Table 4 displays lung cancer deaths by smoking and exposure status, excluding 105 cases with no precise diagnosis. Among smokers or ex-smokers, 11.7% of deaths were due to lung cancer (18/154), while it was 4.4% (4/91) among never-smokers. For those with unknown smoking status, 9.1% of deaths were due to lung cancer, slightly lower than for smokers.

The 4.4% lung cancer death rate among never-smokers (out of 91 deaths) raises questions. A US study [12] found a 0.77 per 1000 person-years incidence of lung cancer in never-smoker men aged 75–79. With 40% never-smokers in this cohort and 90,988 total person-years (54,593 person-years in never-smokers), we estimate more lung cancer cases than the current 4%.

Blue-collar workers (2015 subjects) had 102 lung cancer deaths, about 14 times the 7 deaths among white-collar workers (146 subjects), proportional to their numbers. Lung cancer deaths were 9.6% of all deaths in blue-collar workers (102/1068) and 6.3% in white-collar workers (7/112). The crude mortality ratio of 1.5 indicates lung cancer deaths are 50% more common among blue-collar workers.

The mean and median time to lung cancer were 53.5 and 54.0 years from the start of smoking (range 18.0–78.0 years) and 34.9 and 36.6 years from asbestos exposure (range 0.8–56.6 years) for those who died of lung cancer.

The Cox proportional hazards regression model was used to analyze the association of pleural and lung cancers with asbestos exposure (job title) and tobacco smoke. Table 5 shows the number of cases, hazard ratios (HRs), 95% confidence intervals (CIs), and *p*-values for each covariate. The reference categories are white-collar workers for asbestos exposure and never-smokers for smoking status.

Results indicate an excess risk of pleural cancer in blue-collar workers, though not statistically significant (*p*-value < 0.05). This may be due to limited sample size, exposure misclassification, or the high risk of mesothelioma even at low asbestos exposure levels. Smoking status is strongly associated with lung cancer, with smokers having an HR of 3.26 (*p*-value 0.03), indicating more than double the risk of death compared with never-smokers.

A Monte Carlo simulation with 10,000 iterations was conducted to evaluate the interaction effect between asbestos exposure and smoking status on lung cancer risk. Missing smoking status data were imputed based on the conditional frequency of being a smoker: 40% for white-collar and 40.7% for blue-collar workers. In each iteration, a Cox proportional hazards model was estimated, recording the HR for the interaction between asbestos exposure and smoking.

The simulation results showed mean and median HRs for the interaction of 1.7 and 1.2, respectively, indicating a potential interaction effect. However, the *p*-value of 0.80 and a wide 95% CI (0.25 to 5.98) suggest substantial variability and uncertainty. These findings indicate that more data or larger sample sizes are needed for clearer conclusions.

Following Hosmer and Lemeshow’s methodology, three interaction indicators were calculated: the Relative Excess Risk due to Interaction (RERI), the Attributable Proportion (AP), and the Synergy Index (S). The mean RERI was 0.04, and the median RERI was 0.24, with a 95% CI of −2.07 to 0.99, indicating a neutral interaction effect. The AP mean was 0.14 and its median 0.16, with a 95% CI of −0.72 to 0.89, also suggesting no significant deviation from an additive effect. The Synergy Index mean was 0.92 and its median 0.13, with a 95% CI of −5.11 to 6.88, indicating a potential negative interaction but with considerable uncertainty.

Overall, the analysis suggests no significant interaction effect between asbestos exposure and smoking on lung cancer risk, though the high variability and wide confidence intervals highlight the need for further investigation.

## 4. Discussion

The results of the study of this cohort of workers who were occupationally exposed to asbestos between 1960 and 1979 are in line with what is already known about the risk of mesothelioma due to exposure to this mineral.

In particular,

- The number of cases of mesothelioma far exceeds the number expected in a cohort of workers unexposed to asbestos.
- The risk of developing mesothelioma rises exponentially with the time elapsed since the beginning of exposure.
- The risk of developing mesothelioma does not increase with the duration of exposure but only with the time elapsed since the beginning of exposure.
- The risk of developing mesothelioma does not decrease with the time elapsed since the end of exposure.

More interesting findings are shown in this cohort regarding asbestos exposure, smoking, and lung cancer.

The number of workers dead due to lung cancer is approximately the same as the number of workers dead due to pleural cancer (mesothelioma). This is somewhat expected, as the predominant type of asbestos used was crocidolite, and it is known that in cohorts of workers exposed to this type of asbestos, the ratio of mesothelioma/lung cancer cases is less than 1 [13].

Another interesting finding is that the number of deaths due to lung cancer so far recorded in this cohort is not significantly different from the number expected according to the smoking status of the workers. This is more in line with recent studies on the interaction between tobacco smoke and asbestos exposure which point to a simple additive effect [5]; this means that workers who have developed lung cancer have developed it due to either tobacco smoke or asbestos exposure, but there is no case in which a worker who should have not developed lung cancer has developed it due to the interaction of the two risk factors.

This is corroborated by the fact that the number of cases of lung cancer registered in this cohort is in line with the number expected in a comparable (with regard to smoking habits) population and that the neutral values of the RERI and the AP indicate an absence of significant effects. The positive value of S suggests a possible negative interaction; however, the wide confidence interval indicates considerable variability and lack of precision in the estimates, limiting the conclusiveness of this result.

As all mortality cohort studies, this one has limitations. In studies like this, there are concerns about the precise identification of the cohort (have all the workers exposed been included?), the time between the exposure period and the realization of the study, the reliance on death codes which are well known to be affected by several biases [14], the possibly imprecise retrospective attribution of exposure status and level, and so on.

However, this study also has some strengths. One of them is that the medical records of approximately one-third of the workers were individually checked to extract any useful information (especially smoking status), whereas in registry-based studies, almost nothing is known about each individual worker.

More than one-third of the workers in this cohort are still alive, so in the next iterations of this study, we expect to collect more cases of pleural and lung cancer, thus increasing the power of the statistical models used.

## 5. Conclusions

Our data show a simple additive effect of asbestos exposure and smoking on lung cancer risk and emphasize the importance of considering both asbestos exposure and smoking in occupational health assessments.

These statements reinforce the need for comprehensive occupational health assessments considering both asbestos exposure and smoking history, support public health policies aimed at reducing asbestos exposure and smoking prevalence, and advocate for the continued monitoring of asbestos-exposed populations to inform preventive measures and early interventions.

## Figures and Tables

**Table 1:** Temporal distribution of pleural cancers and mesotheliomas from 1960 onwards.

Calendar Period	Number of Pleural Cancers	Mean Age at Death	Number of Mesotheliomas	Mean Age at Death	Total Cases
1970–1979	1	64.5	-	-	1
1980–1989	12	58.8	-	-	12
1990–1999	16	67.2	2	62.6	18
2000–2009	20	73.1	4	67.9	24
2010–2019	-	-	36	72.0	36
2020–2022	-	-	12	78.4	12
**Total**	49		54		103

**Table 2:** Smoking status of cohort according to job title and smoking habits according to life status, regardless of job title.

Smoking Status	White-Collar Employees n (%)	Blue-Collar Employees n (%)	Total n (%)
**Smokers**	8 (3.0%)	261 (97.0%)	269 (100%)
**Never-smokers**	12 (3.0%)	381 (97.0%)	393 (100%)
**No information**	122 (8.4%)	1329 (91.6%)	1451 (100%)
**Total**	142 (6.7%)	1971 (93.3%)	2113 (100%)
**Smoking Status**	**Deceased** **n (%)**	**Alive** **n (%)**	**Total** **n (%)**
**Smokers**	96 (35.6%)	174 (64.4%)	270 (100%)
**Never-smokers**	167 (42.3%)	228 (57.7%)	395 (100%)
**No information**	1040 (70.1%)	443 (29.9%)	1483 (100%)
**Total**	1303 (60.7%)	845 (39.3%)	2148 (100%)

**Table 3:** Temporal distribution of lung cancers from 1960 onwards.

Year	Number of Lung Cancers	Mean Age at Death (SD)
1960–1969	1	42.8 (±8.4)
1970–1979	8	60.8 (±11.1)
1980–1989	23	64.8 (±7.9)
1990–1999	21	70.2 (±9.8)
2000–2009	25	75.5 (±7.7)
2010–2019	27	75.4 (±9.3)
2020–2022	4	76.7 (±3.5)

**Table 4:** Distribution of lung cancer deaths according to information about smoking status and exposure status (105 deaths excluded from the total count due to the absence of a precise diagnosis).

Smoking Status	Deaths Due to Lung Cancer in White-Collar Workers	Deaths Due to Lung Cancer in Blue-Collar Workers	Total Deaths Due to Lung Cancer	Deaths Due to Other Death Causes	Total Deaths (Any Coded Cause) According to Smoking Status
**Smokers**	0	18	18	136	154
**Never-smokers**	0	4	4	87	91
**No information**	7	80	87	866	953
**Total**	7	102	109	1089	1198
**Total deaths (any coded cause) according to exposure status**	112	1068			

**Table 5:** Association of asbestos exposure and smoking status with pleural and lung cancers.

Death Cause	Asbestos Exposure (Job Title)			Smoking Status		
	N	HR (95% CI)	*p*-Value	N	HR (95% CI)	*p*-Value
**Pleural cancer**	103	1.86 (0.76–4.58)	0.18	20	0.46 (0.19–1.13)	0.09
**Lung cancer**	109	1.42 (0.66–3.06)	0.37	22	3.26 (1.10–9.64)	0.03

## Data Availability

Data presented in this study are available on request from the first authors.

## Appendix A

Collaborators of the Pleural Cancer 2024 Study Group: Yohama Auxilladoras Caraballo Arias, Francesca Graziosi, Paola Caffaro, Francesco Roccuzzo and Samantha Rota.
